# Identification of a HIV-1 circulating BF1 recombinant form (CRF75_BF1) of Brazilian origin that also circulates in Southwestern Europe

**DOI:** 10.3389/fmicb.2023.1301374

**Published:** 2023-11-30

**Authors:** Joan Bacqué, Elena Delgado, Horacio Gil, Sofía Ibarra, Sonia Benito, Isabel García-Arata, María Moreno-Lorenzo, Ester Sáez de Adana, Carmen Gómez-González, Mónica Sánchez, Vanessa Montero, Michael M. Thomson

**Affiliations:** ^1^HIV Biology and Variability Unit, Centro Nacional de Microbiología, Instituto de Salud Carlos III, Madrid, Spain; ^2^Department of Infectious Diseases, Hospital Universitario Basurto, Bilbao, Spain; ^3^Department of Microbiology, Hospital Universitario de Fuenlabrada, Madrid, Spain; ^4^Bioaraba, Microbiology, Infectious Diseases, Antimicrobials and Gene Therapy Research Group, Vitoria-Gasteiz, Spain; ^5^Osakidetza-Basque Health Service, Hospital Universitario Araba, Vitoria-Gasteiz, Spain

**Keywords:** HIV-1, circulating recombinant forms, molecular epidemiology, phylogeny, phylodynamics, recombination, genetic diversity

## Abstract

**Introduction:**

The high recombinogenic potential of HIV-1 has resulted in the generation of countless unique recombinant forms (URFs) and around 120 reported circulating recombinant forms (CRFs). Here we identify through analyses of near full-length genomes (NFLG) a new HIV-1 CRF derived from subtypes B and F1.

**Methods:**

HIV-1 protease-reverse transcriptase (Pr-RT) sequences were obtained by RT-PCR amplification from plasma RNA. Near full-length genome sequences were obtained after amplification by RT-PCR in 5 overlapping fragments. Phylogenetic sequence analyses were performed via maximum likelihood. Mosaic structures were analyzed by bootscanning and phylogenetic analyses of genome segments. Temporal and geographical estimations of clade emergence were performed with a Bayesian coalescent method.

**Results:**

Through phylogenetic analyses of HIV-1 Pr-RT sequences obtained by us from samples collected in Spain and downloaded from databases, we identified a BF1 recombinant cluster segregating from previously reported CRFs comprising 52 viruses, most from Brazil (*n* = 26), Spain (*n* = 11), and Italy (*n* = 9). The analyses of NFLG genomes of 4 viruses of the cluster, 2 from Spain and 2 from Italy, allowed to identify a new CRF, designated CRF75_BF1, which exhibits a complex mosaic structure with 20 breakpoints. All 4 patients harboring CRF75_BF1 viruses studied by us had CD4^+^ T-cell lymphocyte counts below 220/mm^3^ less than one year after diagnosis, a proportion significantly higher (*p* = 0.0074) than the 29% found in other patients studied in Spain by us during the same period. The origin of the clade comprising CRF75_BF1 and related viruses was estimated around 1984 in Brazil, with subsequent introduction of CRF75_BF1 in Italy around 1992, and migration from Italy to Spain around 1999.

**Conclusion:**

A new HIV-1 CRF, designated CRF75_BF1, has been identified. CRF75_BF1 is the 6th CRF of South American origin initially identified in Western Europe, reflecting the increasing relationship of South American and European HIV-1 epidemics. The finding of low CD4^+^ T-cell lymphocyte counts early after diagnosis in patients harboring CRF75_BF1 viruses warrants further investigation on the virulence of this variant.

## 1 Introduction

HIV-1 has a characteristically high recombinogenic potential, which offers some evolutionary advantages to the virus, such as escape from immune responses (Streeck et al., 2008; Ritchie et al., 2014), acquisition of antiretroviral drug resistance (Nora et al., 2007), increased replicative fitness (Gundlach et al., 2000; Moradigaravand et al., 2014; Arenas et al., 2016), and increased viral diversity (Charpentier et al., 2006; Song et al., 2018). The elevated recombination rate of HIV-1 is reflected in the frequent generation of recombinant forms wherever two or more genetic forms circulate in the same population (Nájera et al., 2002). Some HIV-1 recombinant forms have acquired the capacity to spread in the population among epidemiologically-unlinked individuals. These are designated circulating recombinant forms (CRFs) (Robertson et al., 2000), of which around 120 have been reported in the literature (HIV Sequence Database, 2023), with variable epidemiological importance. The number of CRFs is increasing unceasingly, as a consequence of both the generation of new CRFs and the identification of old previously unrecognized CRFs. Their prevalence in the global pandemic has increased, reaching an estimated 17% in a global survey in 2010–2015, where URFs represented around 6% infections (Hemelaar et al., 2020), although a recent systematic literature review estimated the global prevalence of HIV-1 recombinant forms to be 29% (Williams et al., 2023); these percentages could be underestimates, considering that most molecular epidemiological studies are based on the analysis of a small fraction of the viral genome. Among CRFs, those derived from subtypes B and F1 are among the most numerous, of which 19 have been reported in the literature, most of them of South American origin, derived from the F1 strain circulating in Brazil, among which CRF12_BF, widely circulating in Argentina and Uruguay (Thomson et al., 2000, 2002; Carr et al., 2001), is the most prevalent. Five of the CRF_BFs of South American origin have been first identified in Western Europe [CRF47_BF1 (Fernández-García et al., 2010), CRF66_BF1 (Bacqué et al., 2021), CRF89_BF1 (Delgado et al., 2021), and CRF122_BF1 (Cañada-García et al., 2022) in Spain; and CRF42_BF1 in Luxembourg (Struck et al., 2015)], which reflects the increasing immigration from South America to some Western European countries. Here we identify a new CRF_BF of Brazilian origin through near full-length genome analyses of viruses collected in Spain and Italy.

## 2 Materials and methods

### 2.1 Samples

Samples from HIV-1-infected individuals were collected in 14 Spanish regions for molecular epidemiological studies or for antiretroviral drug resistance tests. The study was approved by the Committee of Research Ethics of Instituto de Salud Carlos III, Majadahonda, Madrid, Spain. It did not require written informed consent by the study participants, as it used samples and data collected as part of routine clinical practice and patients’ data were anonymized without retaining data allowing individual identification.

### 2.2 PCR amplification and sequencing

An ~1.4 kb pol fragment in protease-reverse transcriptase (Pr-RT) was amplified from plasma RNA by RT-PCR followed by nested PCR as described previously (Delgado et al., 2015) and sequenced with the Sanger method using a capillary automated sequencer. Near full-length genome (NFLG) sequences were obtained for selected samples by amplification in 5 overlapping segments from plasma RNA and sequenced by the Sanger method, as described (Delgado et al., 2002; Sierra et al., 2005; Cañada et al., 2021). The primers used for PCR and sequencing cover a genome fragment spanning HXB2 nt positions 571–9,509 (~8.9 kb). Newly derived sequences are deposited in GenBank under accessions MK341078, OR466119, OR466120, and OR466121.

### 2.3 Phylogenetic sequence analyses

Sequences were aligned with MAFFT v7 (Katoh and Standley, 2013). Initial phylogenetic trees with all Pr-RT sequences obtained by us were constructed via approximate maximum likelihood with FastTree2 (Price et al., 2010) using the general time reversible evolutionary model with CAT approximation to account for among-site rate heterogeneity (GTR + CAT), with assessment of node support with Shimodaira-Hasegawa (SH)-like local support values (Guindon et al., 2010). Subsequent maximum likelihood (ML) trees with sequences of interest were constructed with W-IQ-Tree (Trifinopoulos et al., 2016), using the general time reversible with free rate (GTR + FR) evolutionary model, with assessment of node support with the ultrafast bootstrap (UFB) approximation approach (Hoang et al., 2018). Trees were visualized with MEGA v7.0 (Kumar et al., 2016). GenBank accessions of sequences used in phylogenetic analyses are shown in Supplementary Table 1.

Mosaic structures were analyzed by bootscanning (Salminen et al., 1995) with SimPlot v1.3.5 (Lole et al., 1999). In these analyses, trees were constructed using the neighbor-joining method with the Kimura 2-parameter model and a window width of 250 nucleotides moving in 20 nt steps. To further determine subtype assignations, potentially recombinant segments identified with SimPlot were phylogenetically analyzed with relevant subtype references by ML with W-IQ-Tree and PhyML v3 (Guindon and Gascuel, 2003) and by Bayesian inference with MrBayes v3.2 (Ronquist et al., 2012). The analyses with PhyML were done using the best-fit substitution model selected by Smart Model Selection (Lefort et al., 2017), with assessment of node support with the approximate likelihood ratio, SH-like procedure (Guindon et al., 2010). The analyses with MrBayes were performed using the GTR + G + I substitution model, running two simultaneous independent runs and 8 chains 2–5 million generations long, ensuring that both runs reached convergence, as determined by an average standard deviation of split frequencies <0.01. In bootscan analyses and in phylogenetic trees of genome segments constructed to determine mosaic structures, a reconstructed B-F1 ancestral sequence was used as outgroup to avoid artifacts caused by distant outgroups (Travers et al., 2004; Thomson and Fernández-García, 2011; Seager et al., 2014; Hill et al., 2022). For this reconstruction, the ML method implemented in W-IQ-Tree was used, with the GTR + FR evolutionary model, and sequences with the following GenBank accessions and subtypes: AB253421, AB253429 (A1); K03455, U21135, U63632, AY173951, AY331295, AY423387, DQ853463, MG365763 (B); AY772699, U46016 (C); AB485656, AF005494, FJ771006, FJ771007, FJ771008, FJ771009, MG365763, MG365768 (F1); AF061641, AF084936 (G); AF190127, AF190128 (H); J: AF082394, EF614151 (J); and DQ373063 (SIVcpz), used as outgroup.

### 2.4 Temporal and geographical estimations of clade ancestors

The times and the locations of the most recent common ancestors (MRCA) of the identified CRF and clusters and subclusters within it were estimated using Pr-RT sequences with the Bayesian Markov chain Monte Carlo (MCMC) coalescent method implemented in BEAST v1.10.4 (Suchard et al., 2018). Before the BEAST analysis, the existence of temporal signal in the dataset was analyzed with TempEst v.1.5.3 (Rambaut et al., 2016), which determines the correlation of genetic divergence among sequences (measured as root-to-tip distance) with time. For the BEAST analysis, 7 codon positions with drug resistance mutations in one or more sequences, as determined by CPR v6.0 (Gifford et al., 2009), were removed from the alignment, since we found that their removal increased the temporal signal of the dataset, as reflected in r^2^ values in the TempEst analysis. The BEAST analysis was performed using the SRD06 codon-based evolutionary model (where the 3rd codon position is in a partition different from the 1st and 2nd positions) (Shapiro et al., 2006). We also specified an uncorrelated lognormal relaxed clock and a Bayesian SkyGrid coalescent tree prior (Gill et al., 2013). The MCMCs were run for 50 million generations. Mixing and convergence were checked with Tracer, ensuring that effective sample size values of all parameters were > 200. We performed runs in duplicate, combining the posterior tree files with LogCombiner v1.10.4. The posterior distribution of trees was summarized in a maximum clade credibility (MCC) tree with TreeAnnotator v1.10.4, after removal a 10% burn-in. MCC trees were visualized with FigTree v1.4.2 (Rambaut^1^). Parameter uncertainty was summarized in 95% highest posterior density (95% HPD) intervals.

### 2.5 Coreceptor usage predicition

Coreceptor usage prediction was done with Geno2pheno (Lengauer et al., 2007), based on the analysis of envelope V3 loop sequences, using the recommendations from the European Consensus Group on Clinical Management of HIV-1 Tropism Testing (Vandekerckhove et al., 2011).

### 2.6 Statistical analyses

Statistical analyses were performed with Fisher’s exact test.

## 3 Results

### 3.1 Identification of a HIV-1 BF1 recombinant cluster segregating from reported CRFs

**Figure 1:**
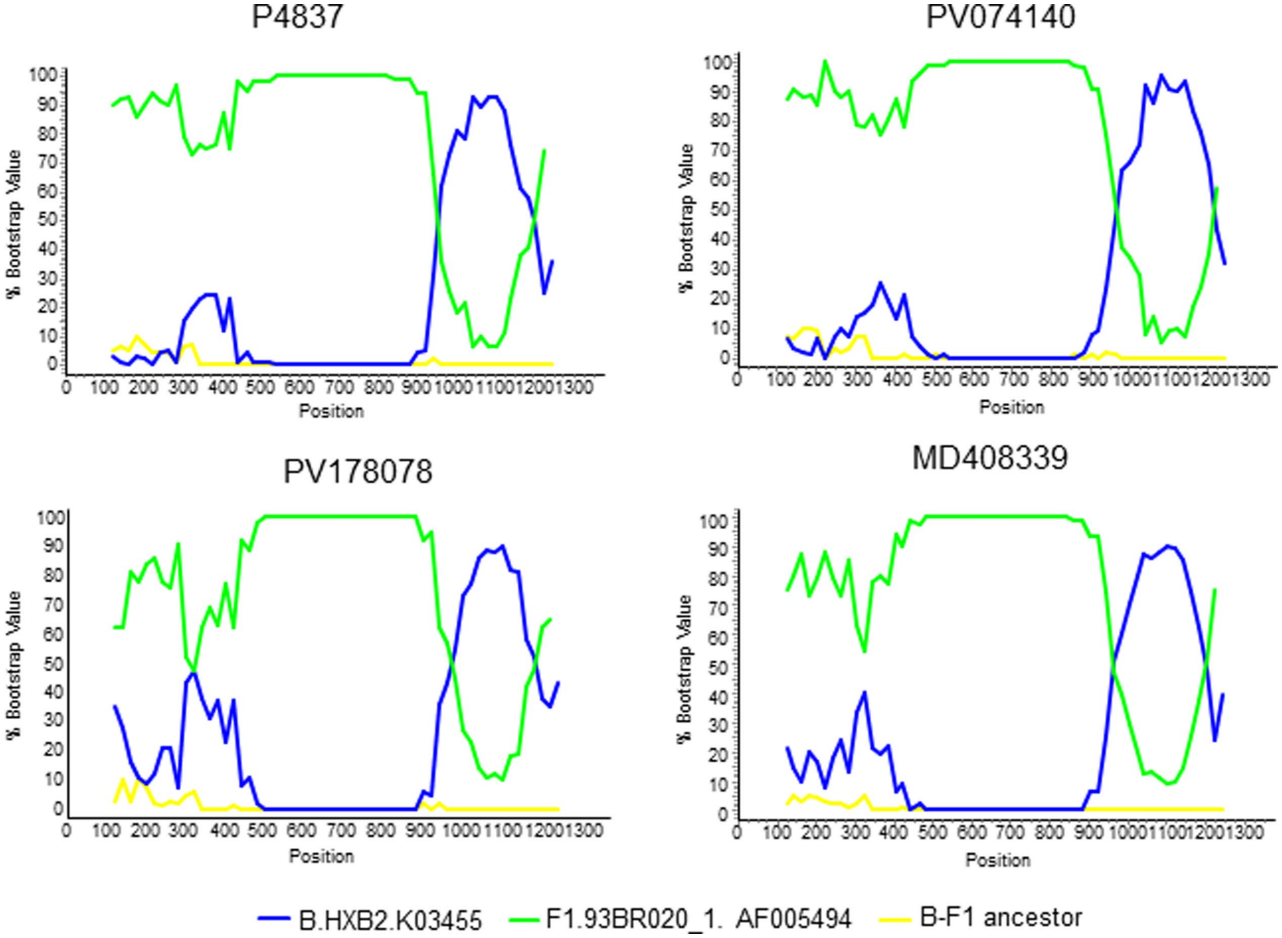
Bootscan analyses of 4 Pr-RT sequences of the BF13 cluster obtained by us. The horizontal axis represents the position from the 5’ end of protease of the midpoint of a 250 nt window moving in 20 nt increments and the vertical axis represents bootstrap values supporting clustering with subtype reference sequences. As outgroup, a reconstructed B-F1 ancestor sequence was used.

**Figure 2:**
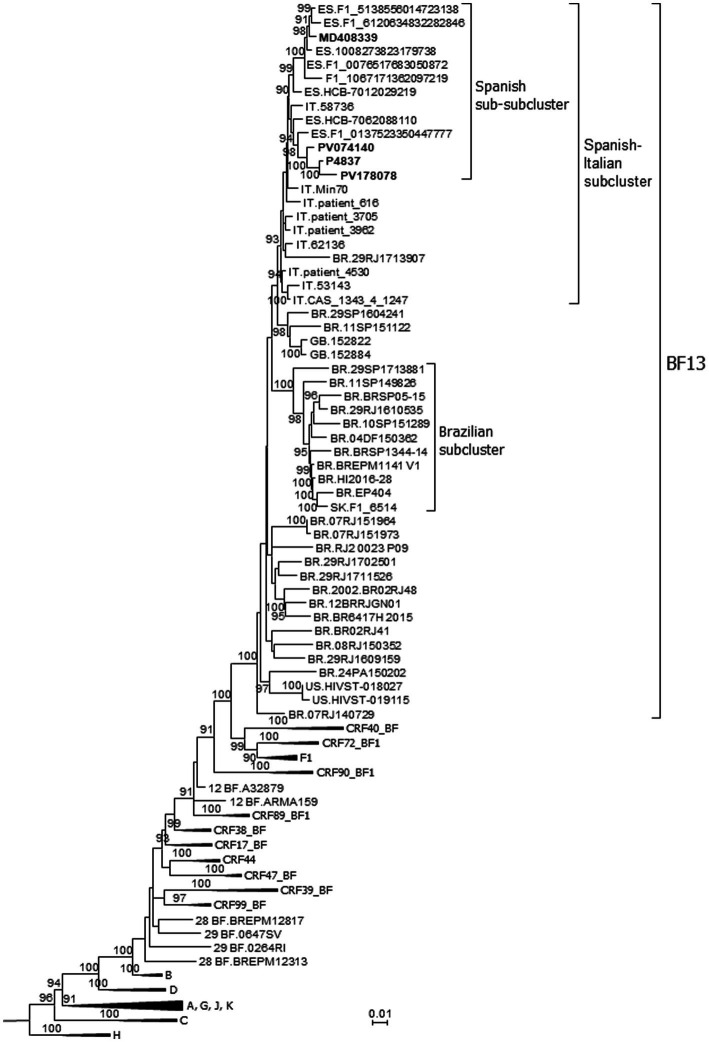
Maximum likelihood tree of Pr-RT sequences of BF13 cluster. References of subtypes and all CRF_BF1s that are BF1 recombinant in Pr-RT are included in the analysis. Names of sequences obtained by us, all collected in Spain, are in bold type. In database sequences, the country of sample collection is indicated before the virus name with the 2-letter ISO country code (ES, Spain; IT, Italy; BR, Brazil; US, USA; UK, United Kingdom; SK, Slovakia). Only ultrafast bootstrap values ≥90% are shown. For viewing purposes, some clades comprising references of subtypes and CRFs are compressed.

In a HIV-1 molecular epidemiological study in Spain, based on analyses of Pr-RT sequences, we found frequent grouping of HIV-1 infections in clusters (Gil et al., 2022), of which 25 were BF1 recombinant branching apart from reported CRF_BFs. One of these clusters, designated BF13 (numbered according to the order of identification), was further analyzed to determine whether it represents a new CRF. In the initial analyses, with sequences obtained by us, BF13 comprised only four individuals, with Pr-RT sequences showing similar recombinant structures, with a predominance of F1 subsubtype and a short subtype B segment around nt positions 1,000–1,200 (numbered from the 5′ end of protease) (Figure 1). Through inclusion in phylogenetic analyses of all Pr-RT sequences from the Los Alamos HIV Sequence database (HIV Sequence Database, 2023) classified as of F1 subsubtype or BF1 recombinant, we found that the 4 BF13 sequences originally identified by us were included in a much larger cluster (for which we kept the BF13 designation) comprising 52 viruses from 6 countries: 11 from Spain, 9 from Italy, 26 from Brazil, 2 from United Kingdom, 2 from USA, and 1 from Slovakia (Figure 2). All 4 individuals from Spain studied by us were native Spaniards, from 3 cities from two regions (Basque Country and Madrid), and all were men who have sex with men (MSM) (Table 1). In the Pr-RT phylogenetic tree, all viruses from Italy and Spain grouped in a subcluster, that also included a Brazilian virus, in which viruses from Spain grouped in a subsubcluster that also included an Italian virus (Figure 2). The bootscan analyses of Pr-RT sequences from the cluster longer than 1.1 kb showed recombinant structures coincident with that of the 4 viruses of BF13 cluster identified by us (Supplementary Figure S1).

**Table 1:** Epidemiological and clinical data of the patients from the BF13 cluster studied by us.

Sample ID	Gender	Transmission route	Country of origin	Province and region of residence	Year of HIV-1 diagnosis	Year of sample collection	CD4^+^ T-cell count (cells/mm^3^)	Plasma viral load (copies/ml)
P4837	M	MSM	Spain	Vizcaya, Basque Country	2017	2017	219	25,300
PV074140	M	MSM	Spain	Vizcaya, Basque Country	2017	2017	96	37,300
PV178078	M	MSM	Spain	Álava, Basque Country	2022	2022	92	22,900
MD408339	M	MSM	Spain	Madrid	2012	2013	142	73,400

### 3.2 Analyses of NFLG sequences

**Figure 3:**
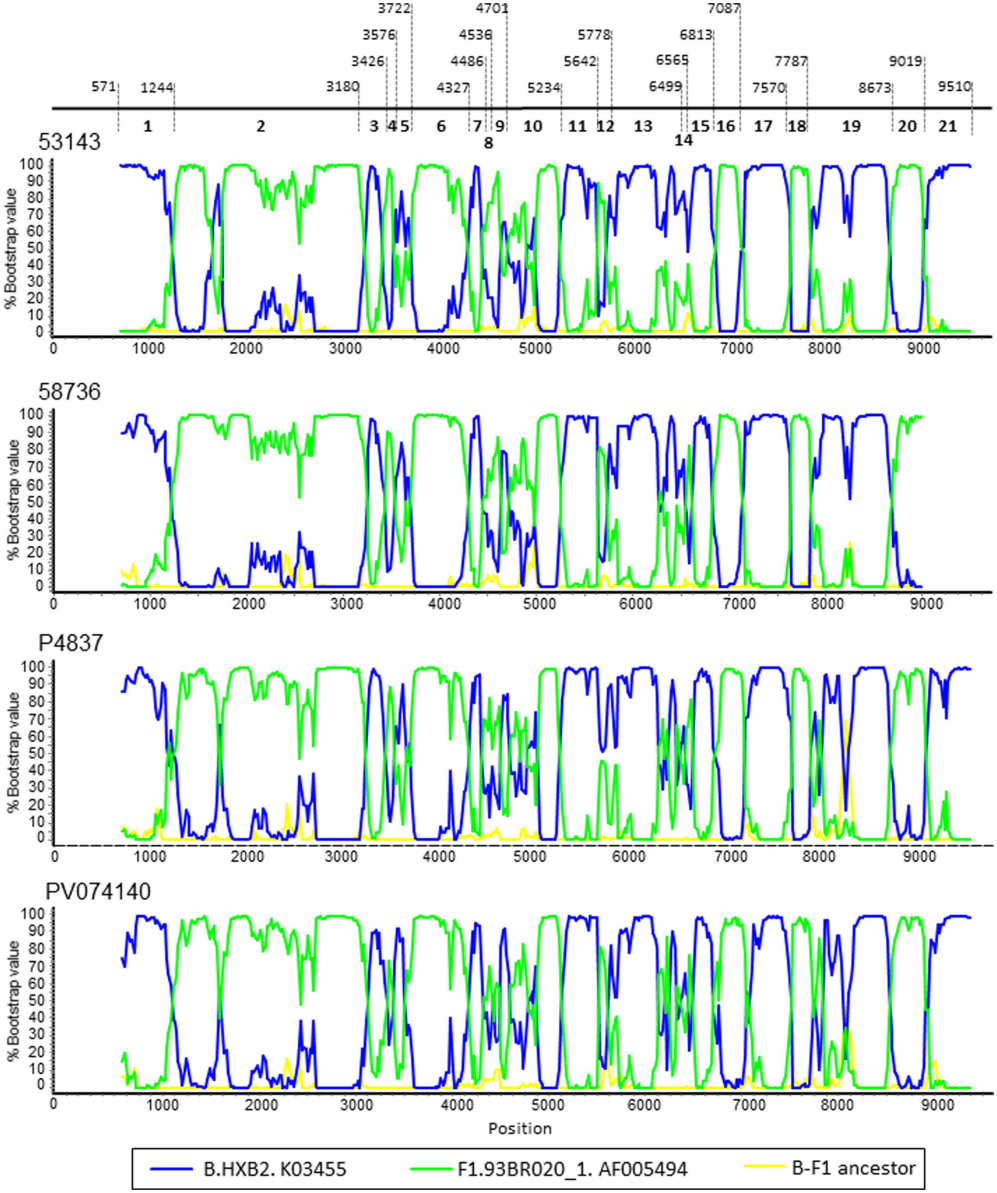
Bootscan analyses of 4 NFLG sequences of viruses from the BF13 cluster, 2 from Spain, obtained by us, and 2 from Italy, obtained by Saladini et al. (2012). The horizontal axis represents the position in the HXB2 genome of the midpoint of a 250 nt window moving in 20 nt increments and the vertical axis represents bootstrap values supporting clustering with subtype reference sequences. As outgroup, a reconstructed B-F1 ancestor sequence was used. Breakpoint positions of segments of potential recombinant origin detected in the bootscan analyses in at least 3 viruses are indicated on top, numbered as in the HXB2 genome. These breakpoints were located in the midpoint positions of the transitions between the 75% consensus sequences of subtype B and of the Brazilian F1 subtype variant.

**Figure 4:**
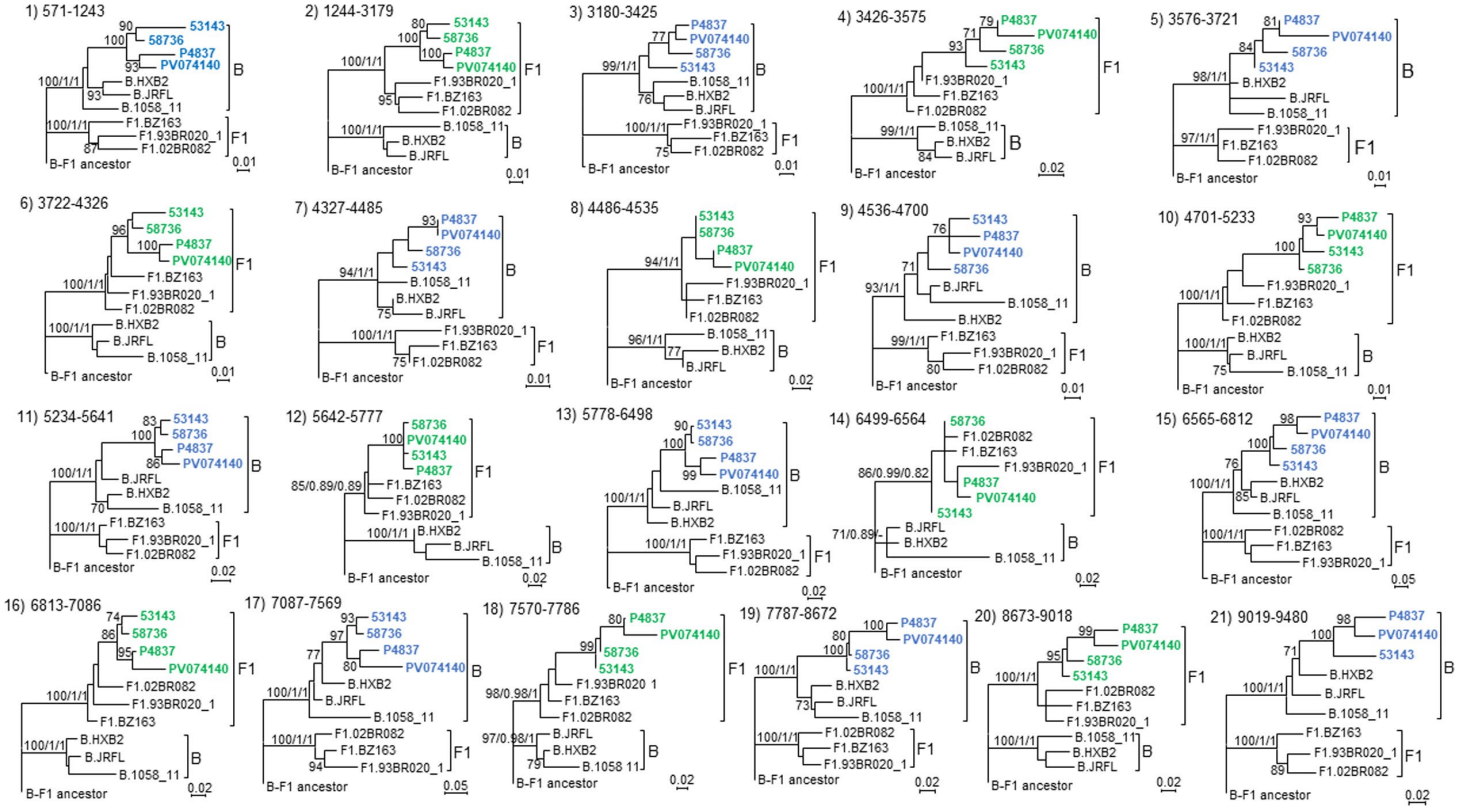
Phylogenetic trees of interbreakpoint genome segments of the NFLG analyzed by bootscanning. The segment number assigned in the bootscan analysis (Figure 3) and the HXB2 positions delimiting the segments are indicated above the trees. Sequence names of BF1 viruses are in blue or green, according to their branching with B or F1 references, respectively, in the corresponding segment. Names of subtype references are preceded by the corresponding subtype name. As outgroup, a reconstructed B-F1 ancestor sequence was used. Node support values of subtype clades are shown in this order: UFB value (IQ-Tree)/ aLRT SH-like support (PhyML)/ posterior probability (MrBayes). For the other nodes, only UFB values, if ≥70%, are shown.

To determine whether the BF13 cluster represents a new CRF, we obtained NFLG sequences of three epidemiologically-unlinked viruses identified by us, from two regions, which were analyzed by bootscanning together with two other viruses from Italy (53,143 and 58,736) from the BF13 cluster whose NFLG sequences were obtained previously by other authors (Saladini et al., 2012). The analyses showed highly similar bootscan plots indicating complex BF1 mosaic structures of two of the Spanish (P4837 and PV074140) and both Italian viruses (Figure 3). ML and Bayesian phylogenetic analyses of the 21 recombinant segments identified in the bootscan analyses in at least 3 of the 4 viruses indicated coincident grouping of all 4 viruses in each segment with B or F1 references (Figure 4). In some apparently recombinant segments seen in the bootscan analyses in only one or two viruses (Figure 3), ML and Bayesian trees failed to support their recombinant origin, since branching with subtype references was not well supported (Supplementary Figure S2). Therefore, these analyses indicated that the 4 viruses exhibited identical mosaic structures.

**Figure 5:**
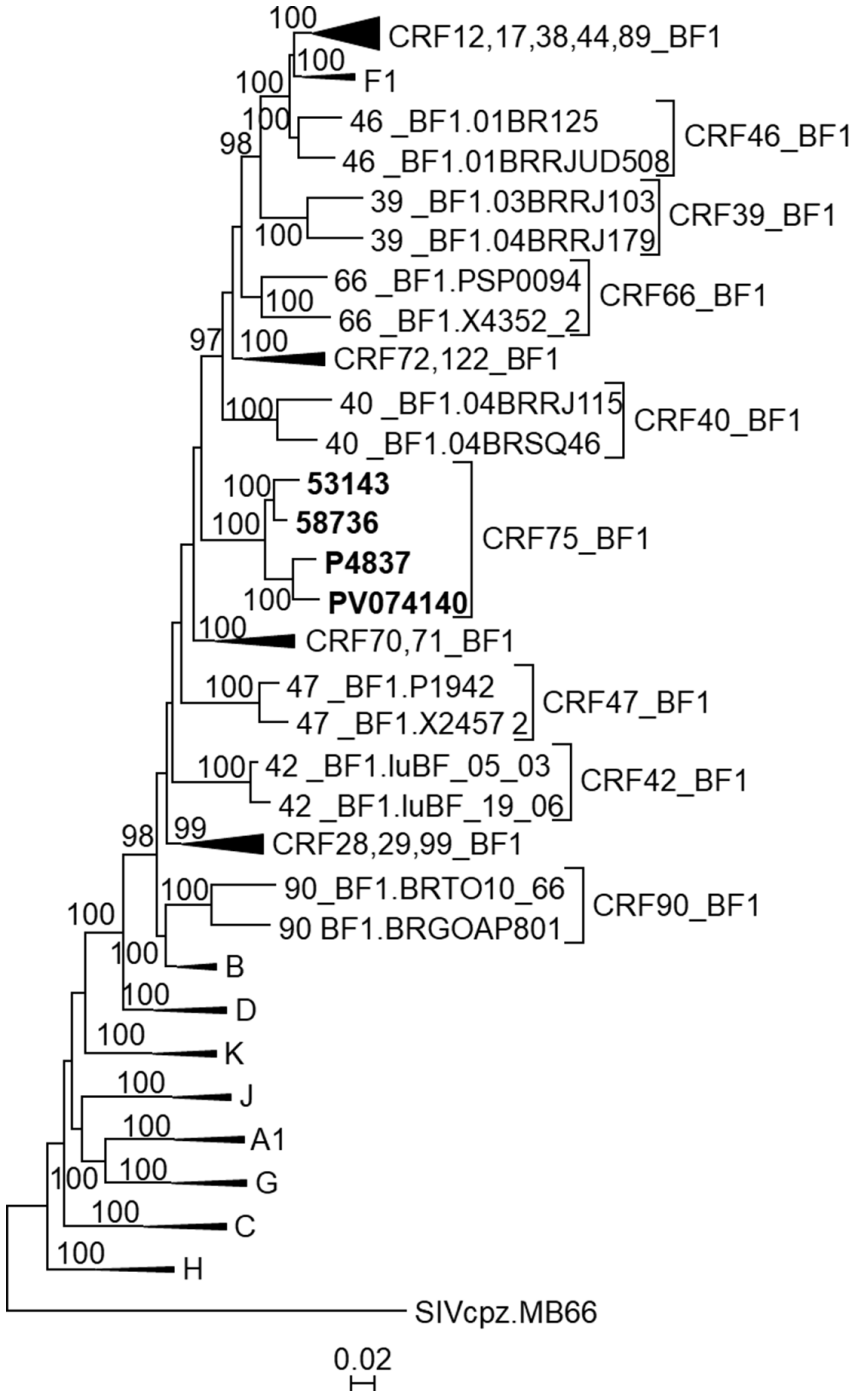
Maximum likelihood tree of NFLG sequences of viruses of the BF13 cluster. References of all published CRF_BF1s and of HIV-1 subtypes are also included in the analysis. The tree is rooted with SIVcpz virus MB66. For viewing purposes, some subtype and CRF reference clades are compressed. Only UFB values ≥90% are shown.

In a phylogenetic tree of NFLGs, the 4 viruses of the BF13 cluster with coincident mosaic structures grouped in a strongly supported clade segregating from previously identified CRF_BFs (Figure 5).

**Figure 6:**
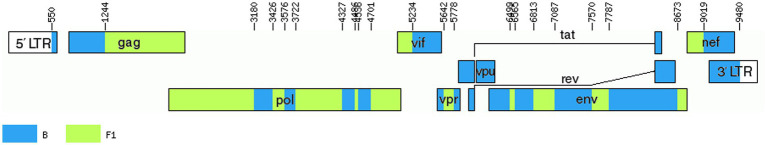
Mosaic structure of CRF75_BF1. Breakpoint positions are numbered as in the HXB2 genome. The drawing was made using the Recombinant HIV-1 Drawing Tool https://www.hiv.lanl.gov/content/sequence/DRAW_CRF/recom_mapper.html.

These results allow us to identify a new CRF, designated CRF75_BF1, which exhibits a complex mosaic structure, with 20 breakpoints, delimiting 11 subtype B and 10 subsubtype F1 segments (Figure 6).

The third NFLG sequence from Spain obtained by us (from patient MD408339) showed a mosaic structure slightly different from the other two in the bootscan analysis, with a short additional subtype B segment in HXB2 positions 6,938–7,086 (Supplementary Figure S3). Since this virus branches in Pr-RT in a subcluster with 5 viruses from the Spanish region of Comunidad Valenciana (Figure 2), it would be interesting to know whether viruses from this subcluster show the same mosaic structure as MD408339, in which case they could represent a new CRF closely related to CRF75_BF1.

The bootscan analysis of a third previously sequenced Italian virus for which around 7 kb from two discontinuous segments were obtained by other authors (Saladini et al., 2012) showed a mosaic structure in the sequenced segments coincident with that of the CRF75_BF1 viruses (Supplementary Figure S3).

### 3.3 Bootscan and phylogenetic analyses of BF1 recombinant sequences from databases related to CRF75_BF1

**Figure 7:**
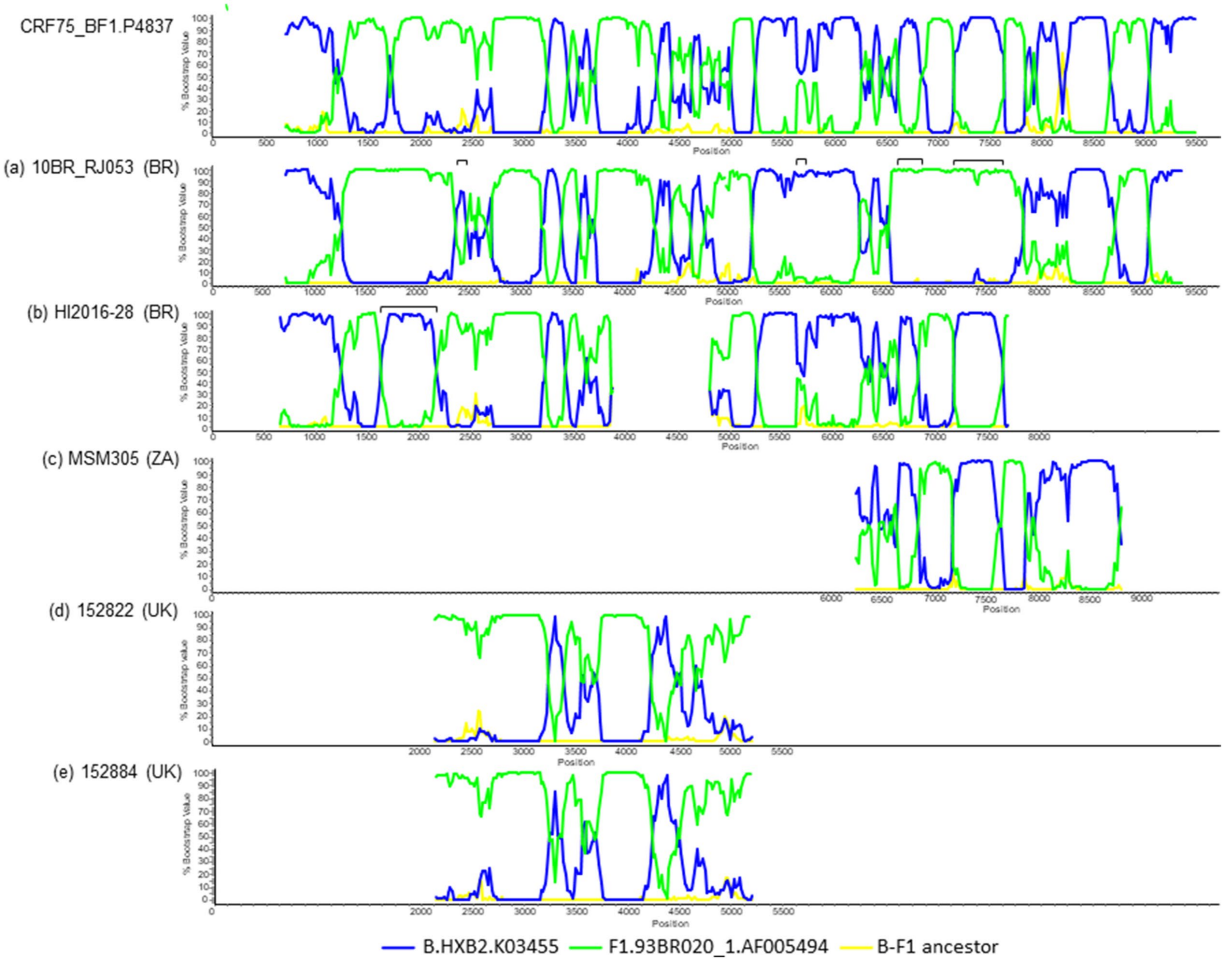
Bootscan analyses of sequences of 5 viruses related to CRF75_BF1. The analyzed viruses are (a) 10BR_RJ053, from Brazil; (b) HI2016-28, from Brazil; (c) MSM305, from South Africa; (d) 152822, from UK; and (e) 152884, from UK. For comparison, the bootscan plot of the NFLG of the CRF75_BF1 virus P4837 is shown on top. The horizontal axis represents the position in the HXB2 genome of the midpoint of a 250 nt window moving in 20 nt increments and the vertical axis represents bootstrap values supporting clustering with subtype reference sequences. As outgroup, a reconstructed B-F1 ancestor sequence was used. Fragments in the analyzed viruses differing in subtype from CRF75_BF1, according to the bootscan analyses and ML phylogenetic trees (Supplementary Figure S4), are indicated as horizontal brackets above the bootscan plots.

Three of the viruses whose Pr-RT sequences branched in the BF13 cluster (HI2016-28, from Brazil, and 152,822 and 152,884, from UK) had additional sequences outside of Pr-RT, that were analyzed to determine their mosaic structures. Through BLAST searches of similar sequences in databases and subsequent phylogenetic and bootscan analyses, we found two additional viruses, from Brazil and South Africa, respectively, that were related to CRF75_BF1. Through these analyses (Figure 7 and Supplementary Figures S4, S5), we found that (1) the Brazilian virus 10BR_RJ053, sequenced in the NFLG sequence (Pessoa et al., 2015), exhibited a recombinant structure highly similar, but not identical, to CRF75_BF1, lacking two B subtype segments in *env* and one F1 subtype segment in *vpr* found in CRF75_BF1 and having an additional subtype B fragment in *pol* (Supplementary Figure S6); (2) the Brazilian virus HI2016-28 (Alves et al., 2017), of which a total of 6.1 kb genome sequence in two discontinuos fragments is available, had a mosaic structure highly similar to CRF75_BF1 but with an additional subtype B fragment in *pol*; (3) the virus from South Africa MSM305 (Middelkoop et al., 2014) had an envelope sequence exhibiting a recombinant structure fully coincident with CRF75_BF1; and (4) the UK viruses 152822 and 152884 had mosaic structures fully coincident with CRF75_BF1 in their available 2.8 kb *pol* sequences. In phylogenetic trees (Supplementary Figure S5), 10BR_RJ053 branched in a basal position relative to the Spanish-Italian CRF75_BF1 cluster, with HI2016-28 branching in an intermediate position, suggesting that 10BR_RJ053 derives from a BF1 recombinant lineage ancestral to HI2026-28 and the Spanish-Italian CRF75_BF1 strain; the South African virus MSM305 grouped with the Brazilian virus HI2016-28, pointing to its probable Brazilian ancestry; and the UK viruses 152822 and 152884 branched basally to the Spanish-Italian CRF75_BF1, which together to their grouping with Brazilian viruses in Pr-RT (Figure 2), also points to their probable Brazilian ancestry.

### 3.4 Temporal and geographical estimations

**Figure 8:**
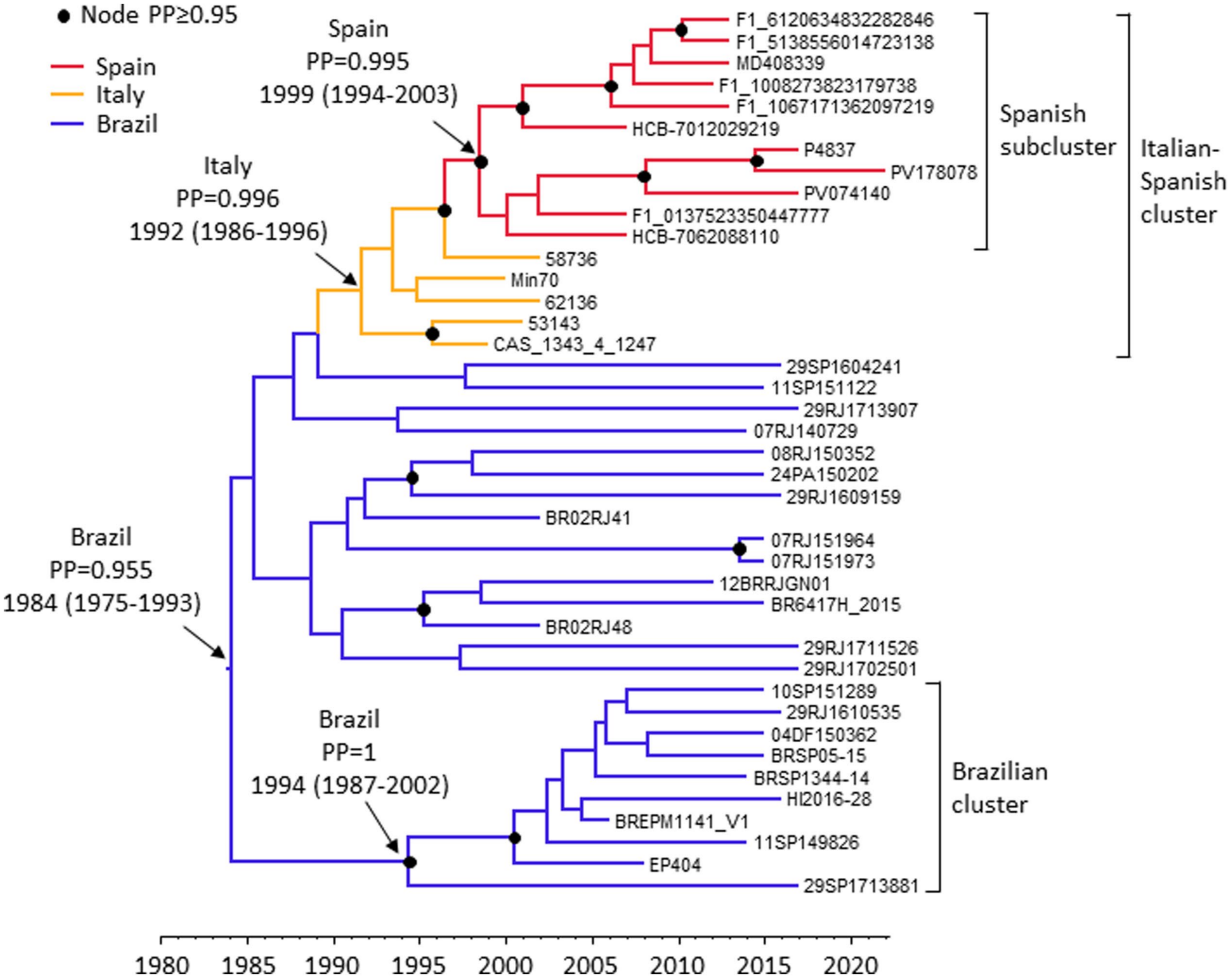
Maximum clade credibility tree of Pr-RT sequences of CRF75_BF1 and CRF75_BF1-like viruses. Branch colors indicate, for terminal branches, country of sample collection, and for internal branches, the most probable location country of the subtending node, according to the legend on the upper left. Nodes supported by PP ≥ 0.95 are marked with filled circles. The most probable countries at the root of the tree and at the nodes corresponding to the Italian-Spanish cluster, the Spanish subcluster, and the largest Brazilian cluster are indicated, together with the PPs supporting each location and the time of the MRCA (mean value, with 95% HPD interval in parentheses).

To estimate the times and places of origin of the clade comprising CRF75_BF1 and related viruses and of the clusters within it, Pr-RT sequences from samples with known collection years where analyzed with a Bayesian coalescent method, implemented in BEAST 1.10.4. Two closely related viruses from United Kingdom and two closely related viruses from USA were excluded from this analysis, as they could derive from sporadic acquisitions in another country where CRF75_BF1 or related viruses circulate. Prior to the BEAST analysis, the TempEst analysis indicated the existence of an adequate temporal signal in the dataset (*r*^2^ = 0.49). The BEAST analysis estimated a substitution rate of 1.34 × 10^−3^/sub/site/yr (95% HPD, 8.52 × 10^−4^-1.86 × 10^−3^). The time of the MRCA of the clade of CRF75_BF1 and related viruses was estimated around 1984 (95% HPD, 1975–1993), and its most probable location was in Brazil (PP = 0.996) (Figure 8). The BEAST analysis also indicated that in Europe, CRF75_BF1 was first introduced in Italy around 1992 (95% HPD, 1986–1996), with subsequent introduction in Spain from Italy around 1999 (95% HPD, 1994–2003).

### 3.5 Association with low CD4^+^ T-cell counts

All 4 patients harboring CRF75_BF1 (or a closely related recombinant in one case) viruses studied by us had low CD4^+^ T-cell counts less than 1 year after HIV-1 diagnosis (3 of them less than 3 months after diagnosis): 219, 96, 92, and 142 cells/mm^3^, respectively. Compared with 3,548 other patients attended at Spanish clinical centers studied by us in the same period in which infections with CRF75_BF1 had been diagnosed (2012–2022), the difference in the proportion of viruses with CD4^+^ T-cell counts ≤220 cells/mm^3^ (100% vs. 29.3%) was statistically significant when analyzed with Fisher’s exact test (*p* = 0.0074).

### 3.6 Coreceptor usage predicitions

Genotypic prediction of coreceptor usage in the 3 viruses studied by us whose sequences of the envelope V3 region were obtained indicated that all were R5 viruses.

## 4 Discussion

The results of this study allow to define an HIV-1 CRF, designated CRF75_BF1, which is the 20th reported CRF derived from subtypes B and F1. Samples harboring CRF75_BF1 or related viruses were collected in 7 countries, but most were from Brazil, Spain, and Italy. Since all 4 individuals from Spain and all 3 from Italy for which data are available were native of the countries of sample collection, it seems that CRF75_BF1 is circulating in both countries among the native population. Transmission mode was men who have sex with men in 4 individuals from Spain and heterosexual in 3 from Italy for which information is available (Saladini et al., 2012).

CRF75_BF1 exhibits a complex mosaic structure, with 20 breakpoints, the largest breakpoint number of any reported CRF (the previous records were for CRF47_BF1 (Fernández-García et al., 2010), CRF18_cpx (Thomson et al., 2005), and CRF56_cpx (Colson et al., 2014), with 16 breakpoints). The highly complex mosaic structures found in CRF75_BF1 and some other CRFs reflect the elevated recombination potential of HIV-1, which can lead to the generation of highly complex recombinant forms through multiple consecutive rounds of recombination (Sierra et al., 2005). In this regard, the identified recombinant strain from Brazil with a mosaic structure slightly less complex than CRF75_BF1 (10BR_RJ053), with 16 breakpoints, 14 of which coincide with those of CRF75_BF1 (Supplementary Figure S6), could derive from an ancestral lineage that led to the generation of CRF75_BF1 through successive recombination events; and the Brazilian virus HI2016-28 (Figure 7B) and Spanish virus MD408339 (Supplementary Figure S3), that show in their available sequences coincidence in mosaic structures with CRF75_BF1, except for the presence of additional subtype B segments, could represent secondary recombinants of CRF75_BF1 with subtype B.

Although no Brazilian CRF75_BF1 virus has been sequenced in the NFLG, we believe that the BF13 cluster as a whole, including Brazilian viruses, most likely corresponds to the CRF75_BF1 clade. We infer this from the following facts and arguments:

1. The Brazilian virus 10BR_RJ053, for which the NFLG sequence is available, has 14 breakpoints coincident with CRF75_BF1 (Supplementary Figures S4, S6) and branches basally to the CRF75_BF1 Spanish-Italian cluster (Supplementary Figure S5). Therefore it seems reasonable to assume that it derives from a BF1 recombinant lineage ancestral to CRF75_BF1.
2. The Brazilian virus HI2016-28, for which sequences of two separate genome fragments summing around 6.1 kb are available, has a mosaic structure almost identical to the Spanish-Italian CRF75_BF1 viruses, with 13 coincident breakpoints, except for a subtype B fragment of around 0.5 kb in *gag* absent from CRF75_BF1 (Figure 7 and Supplementary Figure S4), and in a phylogenetic tree, it branches basally to the Spanish-Italian cluster and closer to it than 10BR_RJ053 (Supplementary Figure S5). It is most parsimonious to assume that the subtype B *gag* fragment of HI2016-28 that is absent from CRF75_BF1 probably derives from secondary recombination of a CRF75_BF1 virus with a subtype B virus, rather than CRF75_BF1 having being generated by secondary recombination of a HI2016-28-like virus, since the lineage ancestral to the Spanish-Italian CRF75_BF1 viruses and HI2016-28 represented by 10BR_RJ053 is of subtype F1 in the mentioned fragment, and B subtype viruses are much more common in Brazil than F1 subtype viruses, which renders much more probable the acquisition through recombination of a B subtype than of a F1subtype fragment. Since HI2016-28 branches within a Brazilian subcluster comprising 11 viruses (Figure 2), the possibility that it represents a new CRF_BF1 derived from secondary recombination of CRF75_BF1 with a subtype B virus should be considered.
3. The South African virus MSM305, for which the full-length envelope sequence (~2.6 kb) is available, has a mosaic structure in the envelope identical to CRF75_BF1 (Figure 7 and Supplementary Figure S4) and groups with HI2016-28 in the ~1.6 kb fragment in which they overlap (Supplementary Figure S5). Therefore, it is probably of Brazilian ancestry.
4. The UK viruses 152822 and 152884, for which *pol* fragments of ~2.8 kb are available, have mosaic structures identical to CRF75_BF1 (Figure 7 and Supplementary Figure S4) and group with two Brazilian viruses in Pr-RT and branch basally to the Spanish–Italian CRF75_BF1 cluster in the 2.8 kb *pol* fragment (Supplementary Figure S5). Therefore, they are also probably of Brazilian ancestry.

Consequently, although no Brazilian NFLG sequences of CRF75_BF1 viruses are available, by joining the fragments of two Brazilian viruses and two viruses of probable Brazilian ancestry showing mosaic structures coincident with CRF75_BF1, a Brazilian BF1 recombinant genome fragment of ~8.2 kb exhibiting a mosaic structure fully coincident with the Spanish-Italian CRF75_BF1 viruses can be reconstructed. Therefore, we can reasonably assume that the BF13 cluster probably represents the CRF75_BF1 clade, although the Brazilian subcluster of 11 viruses observed in Pr-RT (Figure 2) could represent a related CRF derived from secondary recombination of CRF75_BF1 with a subtype B virus. However, to prove or disprove these hypotheses, the analysis of NFLG sequences of Brazilian viruses of the BF13 cluster will be needed.

Two NFLG sequences and around 7 kb of a third genome of CRF75_BF1 viruses from Italy were analyzed in a paper published in Saladini et al. (2012). However, the authors failed to recognize that they represented a new CRF, since they observed small differences in recombinant structures, classifying them as URFs. It is difficult to know the reasons for the discrepant interpretations of analyses, since no bootscan plots and no phylogenetic trees of partial segments were shown in the previous paper.

Phylogeographic analyses place the origin of the clade comprising CRF75_BF1 and related viruses most probably in Brazil around 1984, with diffusion to Italy around 1992 and subsequent migration from Italy to Spain around 1999. The Brazilian ancestry of CRF75_BF1 is consistent with the circulation of subtypes B and F1 in the country, which have given rise to at least 12 reported CRF_BF1s that circulate in Brazil and a 13th (CRF47_BF1) circulating in Spain but originated in Brazil (Hill et al., 2022). The estimated year of origin of the CRF75_BF1 clade is congruent with the earlier estimated origin of the Brazilian F1 strain (around 1977) (Bello et al., 2006) and is similar to the emergence dates of most other South American CRF_BFs reported in the literature (Bello et al., 2010; Ristic et al., 2011; Reis et al., 2017; Bacqué et al., 2021; Delgado et al., 2021; Cañada-García et al., 2022).

CRF75_BF1 and related viruses are found at low prevalences in the countries where they circulate. In Brazil, in a large study on HIV-1 genetic diversity (Gräf et al., 2021), they are found in only 4 (0.05%) of 1,791 samples collected in 2017 (the last year of sample collection of the study). In samples collected in a hospital in central Italy in 1996–2004 (Vicenti et al., 2007), out of 169 sequenced, only 4 (2.3%) are of CRF75_BF1. And among 1,804 sequences from samples collected the region of Comunidad Valenciana, east Spain, in 2004–2014 (Patiño-Galindo et al., 2017), only 6 (0.3%) are CRF75_BF1 or related viruses. This implies that there are CRFs that are circulating at low prevalences that are only detected through the analyses of large numbers of samples, and, consequently, that there are probably many more HIV-1 CRFs than currently known, circulating in undersampled areas. Interestingly, in the cited studies in Brazil and eastern Spain, all CRF75_BF1 or related viruses were classified by the authors as F1 subsubtype viruses, a fact that exposes the limitations of the analysis of Pr-RT sequences for the classification of HIV-1 variants in molecular epidemiological studies.

With the identification of CRF75_BF1, a total of 6 CRFs of South American ancestry have been originally identified in Western Europe (5 CRF_BF1 and 1 CRF_BC) (Fernández-García et al., 2010; Simonetti et al., 2014; Struck et al., 2015; Bacqué et al., 2021; Delgado et al., 2021; Cañada-García et al., 2022). This, together with the propagation of HIV-1 variants of South American origin among the European population (De Oliveira et al., 2010; Collaço Verás et al., 2011; Thomson et al., 2012; Lai et al., 2014; Carvalho et al., 2015; Delgado et al., 2015; Fabeni et al., 2015; Vinken et al., 2019), points to a close relationship between the HIV-1 epidemics in both continents. This may reflect migratory phenomena, particularly in Spain, where around 2.7 million people of South American origin live (Instituto Nacional de Estadística, 2023). In Italy, immigration from South America is substantially lower, but still considerable, with nearly 270,000 South Americans living in this country, around 50,000 of whom are from Brazil (Istat, 2023).

An interesting finding of potential biological relevance is that all 4 patients harboring CRF75_BF1 viruses had CD4^+^ T-cell counts <220/mm^3^ less than 1 year after HIV-1 diagnosis, a proportion that was significantly higher than that found among 3,548 other patients diagnosed in Spain in the same period (29%). Although the number of CRF75_BF1 infections for which CD4^+^ T-cell counts are available is low, this finding suggests the possibility of a greater virulence of CRF75_BF1 that should be investigated in a larger number of patients. In this regard, associations with greater pathogenic potentials have been reported for CRF14_BG (Pérez-Álvarez et al., 2014) CRF19_cpx (Kouri et al., 2015), a CRF01_AE cluster (Song et al., 2019; Ge et al., 2021), and a subtype B cluster (Wymant et al., 2022), and CRF55_01B has been associated with lower CD4 T-cell counts than those found in CRF07_BC infections and higher viral loads than those found in CRF01_AE and CRF07_BC infections (Wei et al., 2021).

The identification and genetic characterization of HIV-1 CRFs may be of interest not only for examining their associated biological properties, but also for vaccine immunogen design, since greater intra-CRF susceptibilities to immune responses have been reported for some CRFs (Mascola et al., 1994; Hraber et al., 2014).

In summary, we have identified an HIV-1 CRF, designated CRF75_BF1, with a highly complex mosaic structure, involving at least 20 recombination events, that originated in Brazil and circulates in Brazil, Italy, and Spain, with sporadic cases detected in 4 other countries from 3 continents. The finding of low CD4^+^ T-cell counts early after diagnosis in the patients infected with CRF75_BF1 studied by us warrants further investigation on its pathogenic associations in a larger number of patients. The results of this and other studies also advocate for the implementation of molecular epidemiological surveillance systems for the rapid detection of the propagation and characterization of HIV-1 variants with potential increased transmission or pathogenic properties.

## Data availability statement

The datasets presented in this study can be found in online repositories. The names of the repository/repositories and accession number(s) can be found below: https://www.ncbi.nlm.nih.gov/genbank/, MK341078; https://www.ncbi.nlm.nih.gov/genbank/, OR466119; https://www.ncbi.nlm.nih.gov/genbank/, OR466120; https://www.ncbi.nlm.nih.gov/genbank/, OR466121.

## Ethics statement

The studies involving humans were approved by Committee of Research Ethics, Instituto de Salud Carlos III, Majadahonda, Madrid, Spain. The studies were conducted in accordance with the local legislation and institutional requirements. The ethics committee/institutional review board waived the requirement of written informed consent for participation from the participants or the participants’ legal guardians/next of kin because the study used samples and data collected as part of routine clinical practice and patients’ data were anonymized without retaining data allowing individual identification.

## Author contributions

JB: Formal analysis, Investigation, Methodology, Writing – review & editing. ED: Conceptualization, Data curation, Formal analysis, Funding acquisition, Investigation, Methodology, Project administration, Supervision, Validation, Writing – review & editing. HG: Data curation, Writing – review & editing. SI: Writing – review & editing. SB: Methodology, Writing – review & editing. IG-A: Writing – review & editing. MM-L: Methodology, Writing – review & editing. ES: Writing – review & editing. CG-G: Writing – review & editing. MS: Methodology, Writing – review & editing. VM: Methodology, Writing – review & editing. MT: Conceptualization, Formal analysis, Funding acquisition, Investigation, Methodology, Project administration, Supervision, Validation, Writing – original draft, Writing – review & editing.

## Footnotes

^1^ http://tree.bio.ed.ac.uk/software/figtree/
