# Supplementary material for: Identification of a HIV-1 circulating BF1 recombinant form (CRF75_BF1) of Brazilian origin that also circulates in Southwestern Europe
Source: Front Microbiol. 2023 Nov 30;14:1301374. doi: 10.3389/fmicb.2023.1301374 (PMC10731470; doi:10.3389/fmicb.2023.1301374)
Supplement: Supplementary file 2 [file Data_Sheet_1.pdf]

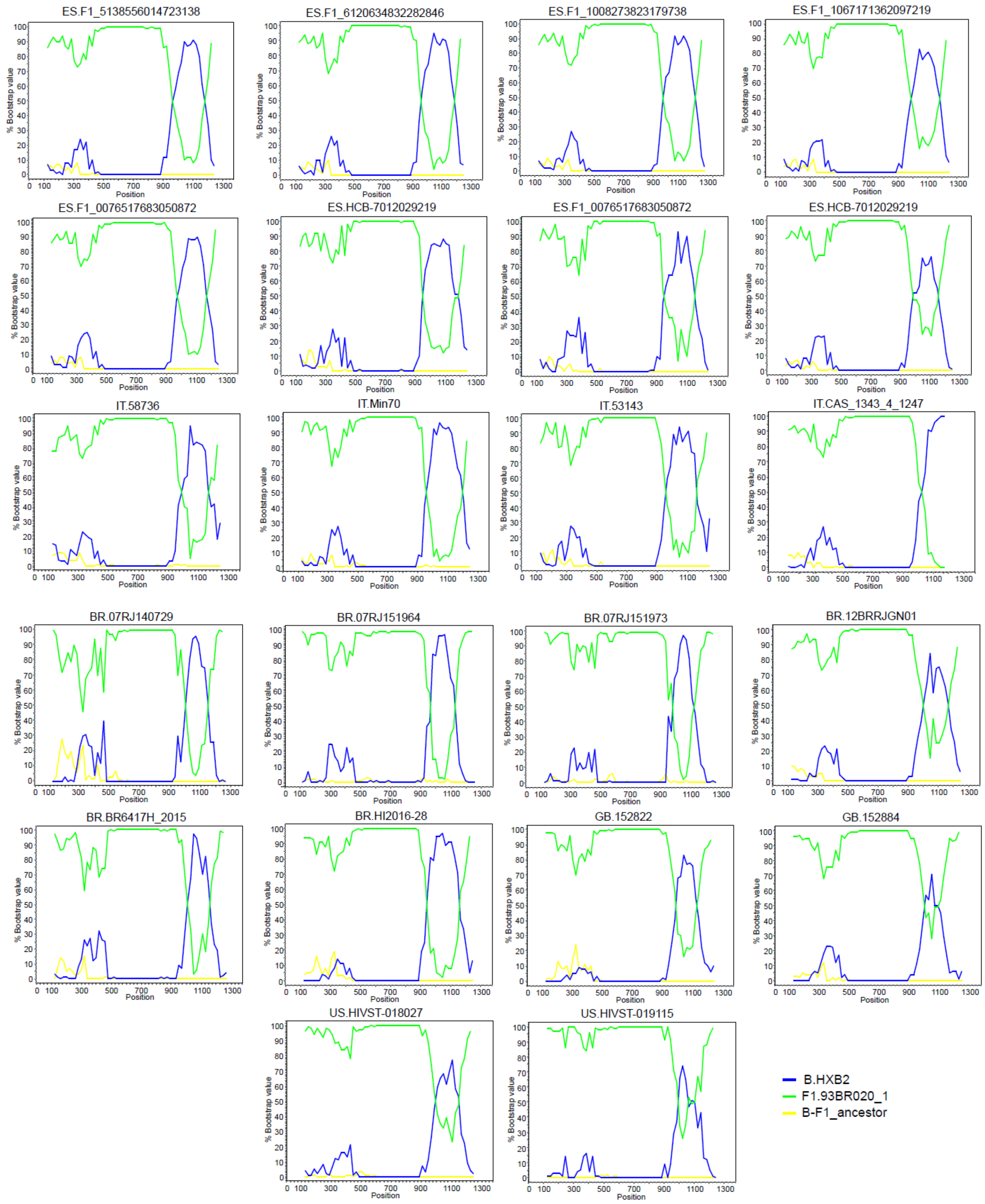

Supplementary Figure 1. Bootscan analyses of Pr-RT sequences of the BF13 cluster from databases. Only sequences >1.1 kb were analyzed. The horizontal axis represents the position from the 5' end of protease of the midpoint of a 250 nt window moving in 20 nt increments and the vertical axis represents bootstrap values supporting clustering with subtype reference sequences. As outgroup, a B-F1 ancestor sequence, reconstructed with IQ-Tree, was used, to avoid artefacts caused by distant outgroups (Hill et al., 2022).
