# Supplementary material for: Identification of a HIV-1 circulating BF1 recombinant form (CRF75_BF1) of Brazilian origin that also circulates in Southwestern Europe
Source: Front Microbiol. 2023 Nov 30;14:1301374. doi: 10.3389/fmicb.2023.1301374 (PMC10731470; doi:10.3389/fmicb.2023.1301374)
Supplement: Supplementary file 3 [file Data_Sheet_2.PDF]

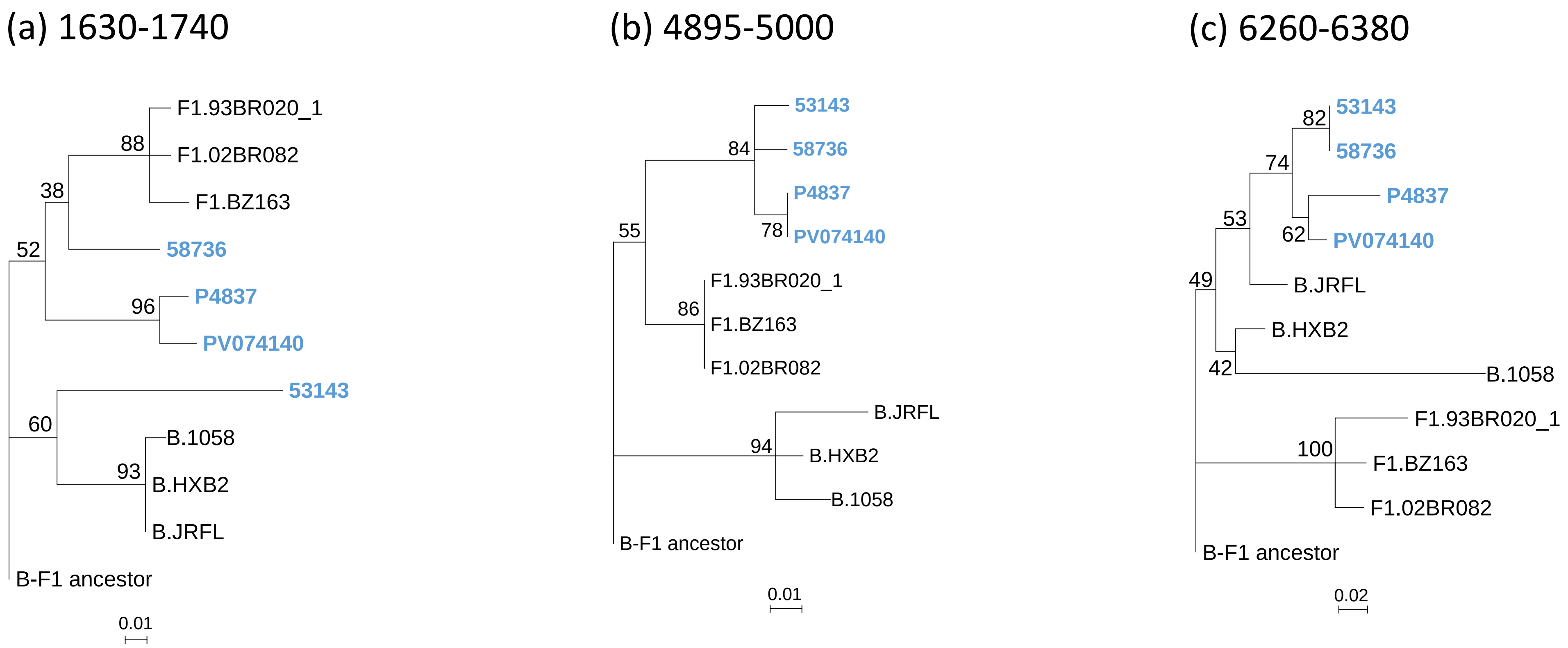

Supplementary Figure 2. Phylogenetic trees of genome segments of the 4 NFLG sequences analyzed by bootscanning (Fig. 3) which appeared to be of recombinant origin in only one or two viruses. The HXB2 positions delimiting the analyzed segments are indicated above the trees. Sequence names of BF1 viruses are in blue. Names of subtype references are preceded by the corresponding subtype name. As outgroup, a B-F1 ancestor sequence, reconstructed with IQ-Tree, was used to avoid the artefacts caused by distant outgroups (Hill et al., 2022). Node support values indicate UFB values obtained with IQ-Tree.
