# Supplementary material for: Identification of a HIV-1 circulating BF1 recombinant form (CRF75_BF1) of Brazilian origin that also circulates in Southwestern Europe
Source: Front Microbiol. 2023 Nov 30;14:1301374. doi: 10.3389/fmicb.2023.1301374 (PMC10731470; doi:10.3389/fmicb.2023.1301374)
Supplement: Supplementary file 4 [file Data_Sheet_3.PDF]

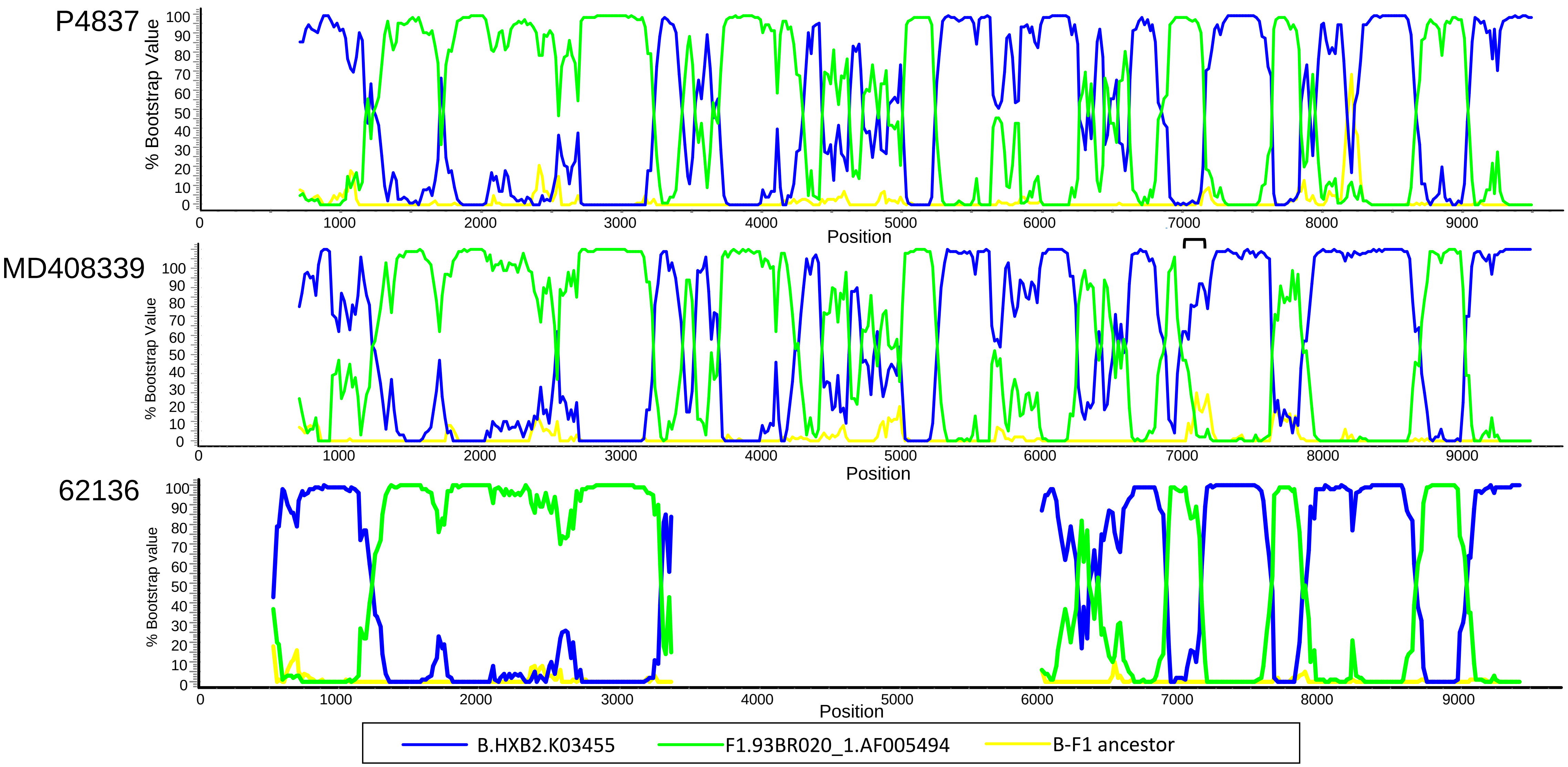

Supplementary Figure 3. Bootscan analyses of genome sequences of the Spanish virus MD408339 and the Italian virus 62136. For comparison, the bootscan plot of the NFLG of P4837 is shown above. The horizontal axis represents the position in the HXB2 genome of the midpoint of a 250 nt window moving in 20 nt increments and the vertical axis represents bootstrap values supporting clustering with subtype reference sequences. As outgroup, a reconstructed B-F1 ancestor sequence was used. A horizontal bracket on top of the MD408339 plot indicates a fragment where MD408339 appears to differ in subtype from P4837.
