# Supplementary figures and images for: Identification of a HIV-1 circulating BF1 recombinant form (CRF75_BF1) of Brazilian origin that also circulates in Southwestern Europe
Source: Front Microbiol. 2023 Nov 30;14:1301374. doi: 10.3389/fmicb.2023.1301374 (PMC10731470; doi:10.3389/fmicb.2023.1301374)

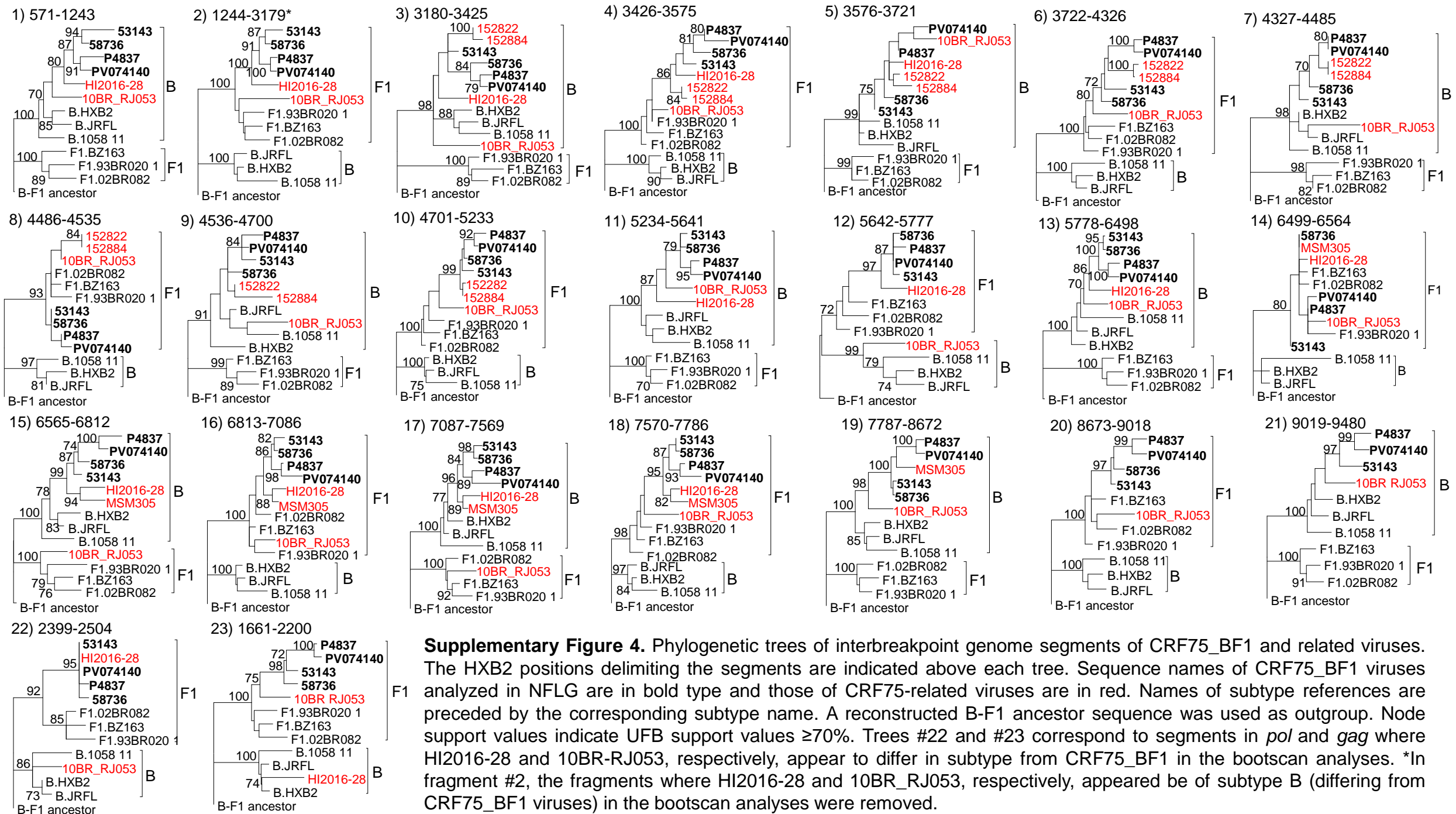

Supplement: Supplementary file 5 [file Data_Sheet_4.PDF]
