# Supplementary material for: Identification of a HIV-1 circulating BF1 recombinant form (CRF75_BF1) of Brazilian origin that also circulates in Southwestern Europe
Source: Front Microbiol. 2023 Nov 30;14:1301374. doi: 10.3389/fmicb.2023.1301374 (PMC10731470; doi:10.3389/fmicb.2023.1301374)
Supplement: Supplementary file 6 [file Data_Sheet_5.PDF]

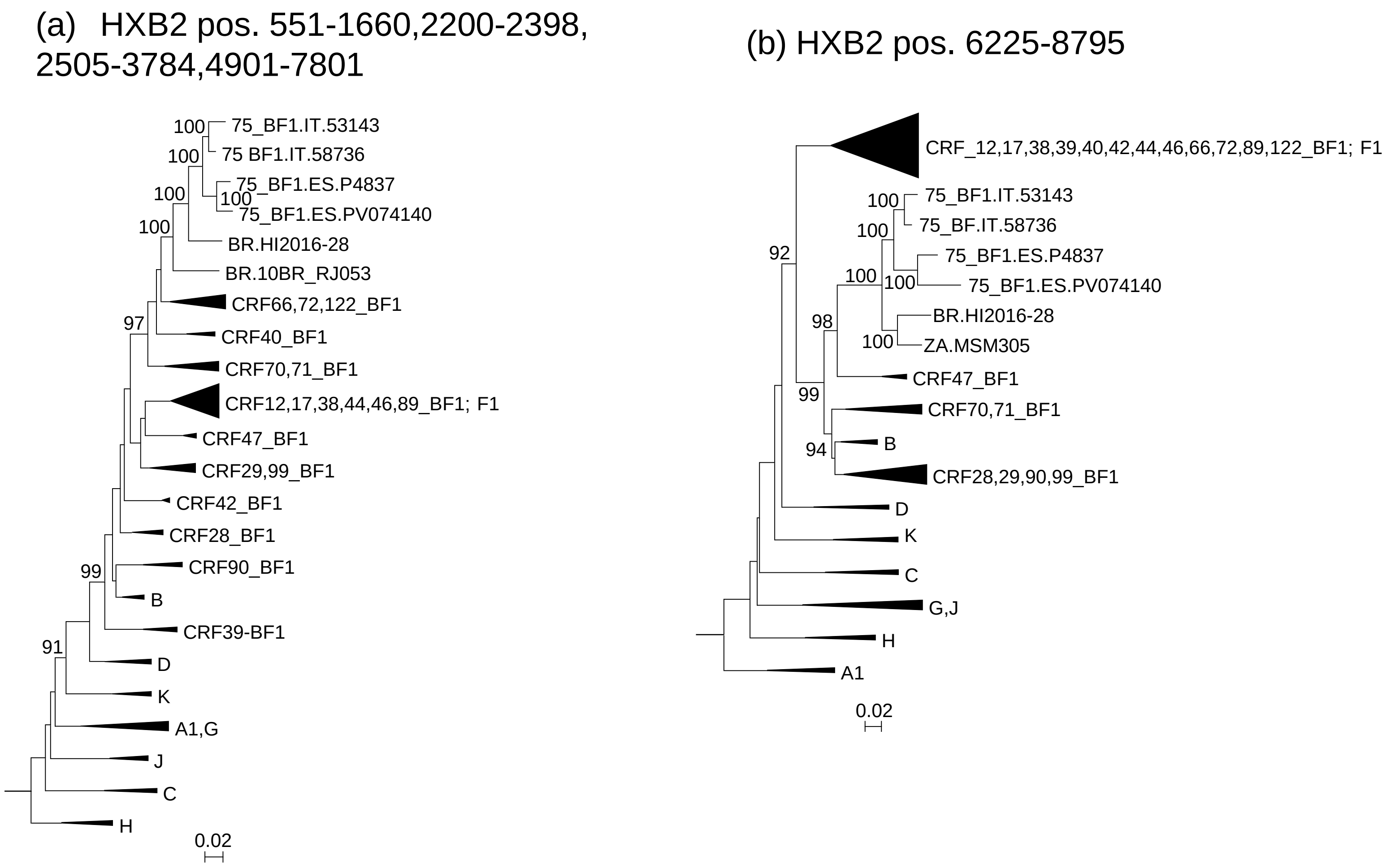

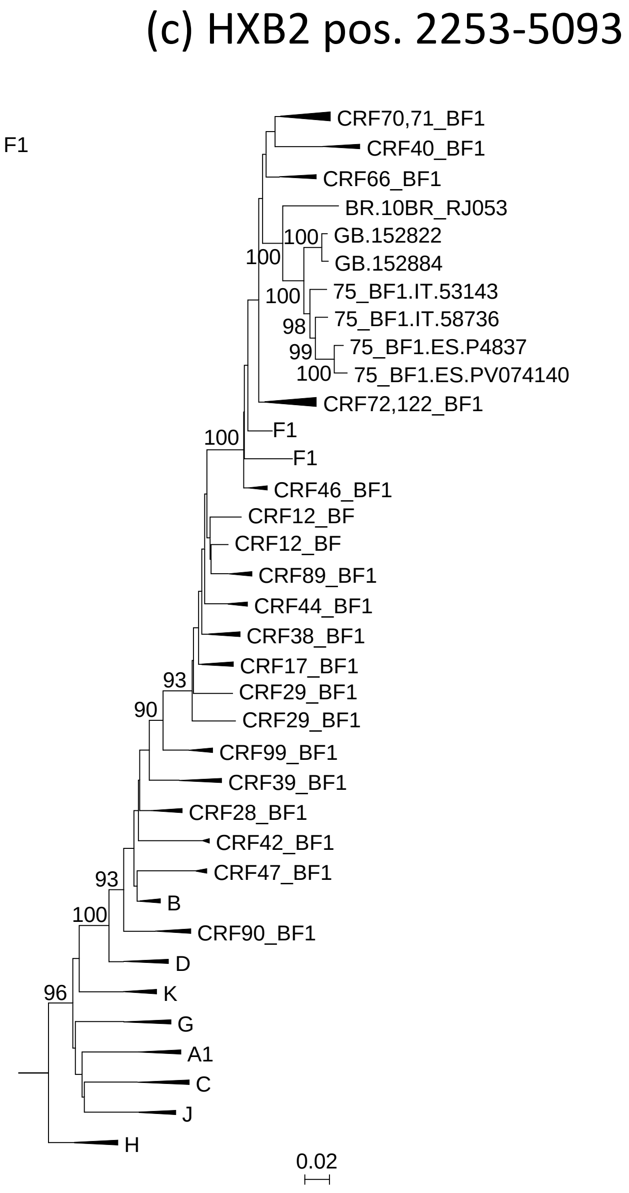

Supplementary Figure 5. Phylogenetic trees showing the relationship of BF1 viruses from (a) Brazil (10BR_RJ053 and HI2016-28), (b) South Africa (MSM305), and (c) United Kingdom (15822 and 15884) with the Spanish-Italian CRF75_BF1 cluster; in (b), the relationship between MSM305 and HI2016-28 is also seen. HXB2 positions of the analyzed segments are shown above each tree. In (a) fragments differing in subtype between 10BR_RJ053 or HI2016-28 and CRF75_BF1 were removed. For viewing purposes, clades of CRF_BF and subtype references are compressed. Trees were constructed with IQ-Tree and only node UFB values ≥90% are shown, except for the nodes of compressed clades, whose UFB values (in all cases >95%) are omitted. Trees were rooted with SIVcpzMB66.
