# Supplementary material for: Identification of a HIV-1 circulating BF1 recombinant form (CRF75_BF1) of Brazilian origin that also circulates in Southwestern Europe
Source: Front Microbiol. 2023 Nov 30;14:1301374. doi: 10.3389/fmicb.2023.1301374 (PMC10731470; doi:10.3389/fmicb.2023.1301374)
Supplement: Supplementary file 7 [file Data_Sheet_6.PDF]

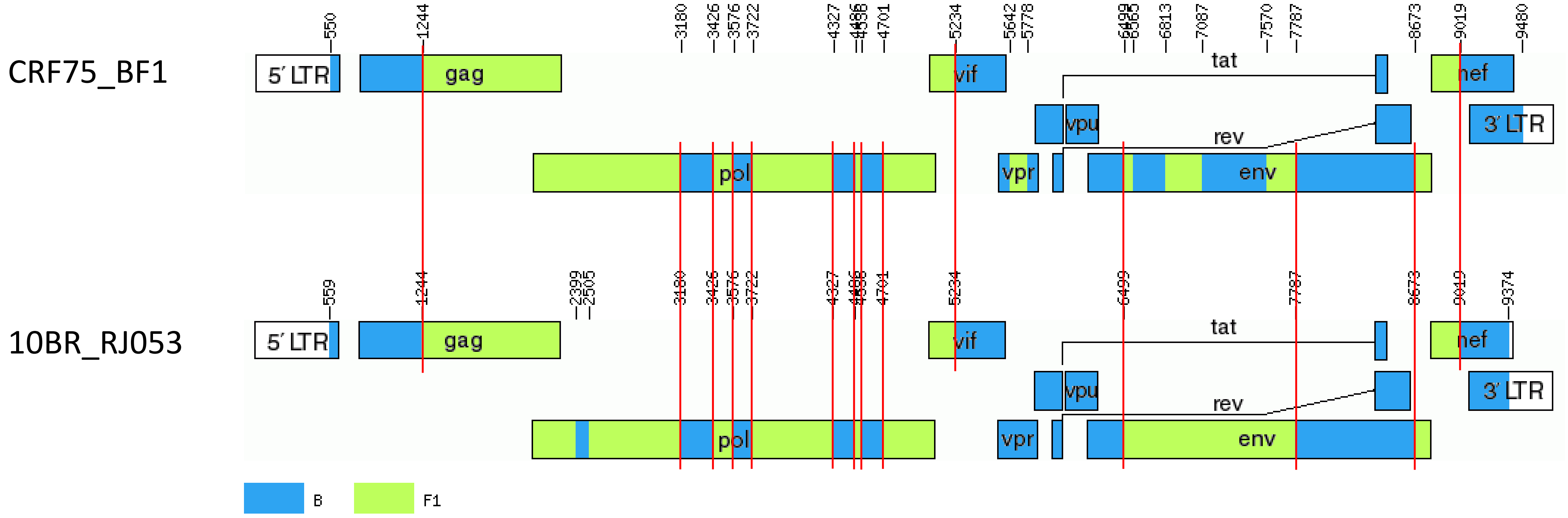

Supplementary Figure 6. Mosaic structure of 10BR_RJ053. Breakpoint positions are numbered according to the HXB2 genome. For comparison, the mosaic structure of CRF75_BF1 is placed above, with vertical lines indicating coincident breakpoints. The drawing was made using the Recombinant HIV-1 Drawing Tool https://www.hiv.lanl.gov/content/sequence/DRAW_CRF/recom_mapper.html
